# Ethics of Consultation1Read at the semi-annual meeting of the Pennsylvania State Veterinary Medical Association, Scranton, September, 1899.

**Published:** 1899-12

**Authors:** Jacob Helmer

**Affiliations:** Scranton, Pa.


### ETHICS OF CONSULTATION.[1]

By Jacob Helmer, D.V.S.,
SCRANTON, PA.

The idea of consultation is based upon confidence. It is a beautiful as well as a practical idea. It presupposes sincerity and virtue. Without morality for a foundation the art of consultation is lost. Knowledge is only of secondary importance in the philosophy of this art, around which cluster every human virtue. Right consultation is a high test of character. It opposes all that is sinister and mercenary. It calls for all that makes one true to himself and others. "Be true to others, to yourself be true," is its motto. We rise in public esteem just in proportion as we fulfil this ideal. The dangers which have threatened our profession, such as the failure to secure proper legislation, the persistence of the empiric, the stolid indifference of the public to our true social status, are gradually disappearing before the growth of the sense of duty and justice to the public and also to each other, as well as the increase of scientific knowledge of our art.

Since duty and justice to the client and one's self is a basic element of success, we may consider circumstances which do and do not demand a consultation. If the case in the veterinarian's charge is not fully understood and the therapeutical treatment has not yielded favorable results; if both the diagnosis and treatment are open to question; if surgical interference be desired and it is not known how to perform the necessary operation; if skilled assistance be required in order that every surgical detail may receive proper attention; if the physician has lost confidence in himself or enthusiasm in the case; if the consciousness exist that everything is being done that it is possible to do, but the client is not quite satisfied, under these and similar circumstances it becomes the duty of the physician to ask for a consultation. On the other hand, where the doctor has carried the case to the extreme limit and death is impending; where no one's help would avail, and where to call for help under such circumstances is but to embarrass the consulting physician by forcing from him the confession that nothing can be done, especially if the client has confidence in the physician called—in other words, if the motive of the attending physician was mercenary—under such conditions it is not right to

[1] Read at the semi-annual meeting of the Pennsylvania State Veterinary Medical Association, Scranton, September, 1899.

call for a consultation; it is not right to divide the responsibility on a dead patient unless the owner desires it while fully understanding that the patient is considered beyond help.

But consultation is primarily for the benefit of the individual under treatment. Whether this idea applies to veterinary patients as forcibly as to human ones is a question left to the good nature of the attending physician. Each one must decide for himself the consideration due to an animal for its life's sake, apart from money value. Humble though that life may be, it is the only one the animal has. Its right to life as much as we to ours cannot be disputed, and when life is gone it cannot be restored. A human being can assist himself, but an animal is helpless. Its very helplessness and dependence, associated with its usefulness and faithfulness, should be sufficient to stimulate in every human heart consideration for the animal's life, comfort, and happiness. The experience of our animal friends is akin to our own.

But it is not always the attending physician who first desires a consultation. Where the latter cannot see any reason for such a proceeding, members of the family or one of the friends may influence the client toward the idea. In such an event it is happy if the physician anticipates the situation and voluntarily signifies his willingness, although he may not consider it necessary, and plainly expresses himself to that effect. In the choice of a consulter there should be no friction. If the client desires one with whom it would be impossible for the attending physician to consult, the latter should promptly make this plain. In all cases the client should defer to the wishes of his physician. It is natural with all concerned that this should be done. It would be an extreme case where the client would not accept any of those named by the one in attendance.

When the consulting physician has been chosen it should be the privilege of the attending physician to issue the invitation, otherwise it might reflect upon his good-will toward all the parties concerned. The interest of the client would not be conserved when the harmony of the situation was disturbed by the suspicion of bias, favoritism, or dissatisfaction. While it is the privilege of the attending physician to call or bring the chosen consulter, it is not his prerogative to discuss the merits or demerits of the case before the patient is reached. It is more creditable not to allude to the subject until the consultation actually begins. The only exception to this would be an emergency case, in which whatever was done would have to be done quickly.

But once in the presence of the patient, the people concerned having recognized each other, the consulting physician should at once begin to investigate the case; the attending physician will give the history and answer all questions, in order that the consulting physician may have the help and data required to assist him in making a diagnosis; then will naturally follow the discussion of the treatment that has been used and its results. In all this the doctors should not be hampered by anybody's immediate presence. The opinion of the consulting physician having been formed, it will be delivered to the client by the attending physician, together with any recommendations that may be offered. Anything concerning the nature and progress of the malady, the prognosis, etc., may now be fully commented upon. The remedies employed should not be disclosed, since none but the physician is supposed to know anything about the reasons for the employment of certain drugs or medicines. Any discussion that may arise through lack of agreement on the case should not be indulged in in the presence or hearing of the client, who cares only for the opinion as to results, whether favorable or unfavorable. Any symptom of trouble discerned by him tends to create doubt and will weaken his confidence. The consultation should be made pleasant for the client, even though friction may exist elsewhere.

The fee of the consulting physician is paid by the client. It should be more than an ordinary one. The fee may have been fixed by a rule of the medical society or may have been agreed upon by the parties concerned. As a rule, the fee should be paid when the consultation is closed. The fee of the consulting physician remains the same as before.

When called in consultation, observe punctuality. Failure to meet means postponement. There is no definite rule as to the length of time one physician should wait for another, this being a matter entirely governed by circumstances; but a half hour should be sufficient. If the attending physician be the delinquent, the consulting physician should not advise in his absence or commit himself; but if the latter cannot avoid examination of the patient, he must put his ideas in writing and seal them, for the benefit of his colleague. Where the consulting physician fails to keep his engagement the attending one will continue the case until the consultation can be held.

In consultation avoid protracted arguments, especially over matters of minor importance—matters of mere theory anyway. Agree quickly on the main points upon which the importance of the case depends. The conversation during a consultation should be considered secret. After-insinuation that any part of the treatment did not receive sanction is despicable. The consulting physician should esteem his colleague as himself, hence refrain from insinuation or double meaning, as well as avoid any look or action which is only another way of telling the client the case has not been properly treated, and by such means, combined with ingenious language, endeavor to ingratiate himself into favor with the client, to the disadvantage of the attending physician. True physicians depend upon qualification, not intrigue, for success.

When a simultaneous call is given several physicians the one arriving first is entitled to treat the case. If it be necessary to call another during the absence of the attending physician, said call does not entitle him to continue the treatment of the case. Subsequent work is due the regular physician. When called to see a patient under the care of another physician a consultation should be demanded. Influences should not be used to have another discharged. When in a difficult case a decided difference of opinion exists it is permissible to call in a third physician; but if the consulting physician feels that due consideration and respect for his opinion is not entertained he should retire from the case promptly.

There are circumstances under which a consultation should be declined. An educated and progressive physician should not put himself in position to consult with one of an opposite character. It is not his business to educate inferior men; the chances are ten to one he will receive no credit for it. He should, therefore, decline to consult with anyone whom he would not call to consult with him. A physician should never consult with a charlatan or one who practises surreptitiously, nor with one who has no knowledge of the sciences upon which the practice of medicine is based. It occasionally occurs, especially in a country practice, that the veterinarian meets one who has charge of the case to which he is called. This man should be treated like a gentleman. Approve of all in his treatment that you can while making the changes deemed necessary for the recovery of the patient. The client will comprehend that it is better to employ a qualified than an unqualified man. If the case cannot recover, remember that the owner is as much to blame as the empiric, who simply did the best he knew how. Remember that to quarrel is to lower your dignity as well as that of the profession. It is not a question of superiority of name, but of knowledge. The latter is always modest. It makes itself felt in silence. All there is to be gained in the case can be done by silence apart from showing knowledge which everybody respects. The physician, instead of creating unpleasant feelings, should win from the attendant the confession that he was simply doing the best he knew how in the absence of a better man. The physician who cannot do this has much to learn.

Who is prepared to say what part of the weakness of our profession is not caused by unfitness to do this work? Public opinion looks into the heart of things. It has its hand ever upon the pulse of character; it sifts the wheat from the chaff, the gold from the dross. Public opinion influences legislation. The latter is its legitimate child. When otherwise secured it is illegitimate. We should, therefore, influence public opinion favorably as the most rapid means of raising the standard of our profession as well as ensuring pecuniary success.

In the art of consultation the physician has before him a series of duties—duty to the consulting physician, the client, the patient, the profession, and, lastly, his duty to himself. In the proper performance of these duties mental and moral qualities of a high order are demanded—qualities which we cannot afford to set aside for selfish motives. Even though to-day it might appear wise and beneficial to do so, to-morrow will discover the weakness and lessen the value of an advantage dishonestly gained. This brings us back to where we began, that consultation is based upon confidence, sincerity, and virtue, without which it fails to be an art.

